# Corrigendum

**DOI:** 10.1177/2333794X18804903

**Published:** 2018-09-20

Azevedo L, Jay A, Lorenzana A, et al. Case Report of an Adolescent Male With Unexplained Pancytopenia: GATA2-Associated Bone Marrow Failure and Genetic Testing. *Glob Ped Hea*. 2017;4. doi:10.1177/2333794X17744947

The above article was published in volume 4 of *Global Pediatric Health*, with the second author, Rofida Nofal, omitted.

Dr. Rofida Nofal wrote the case report section; including patient history, physical exam findings, work up, case progression and follow up, as well as contribution to the background.

The final authorship for the article is:

Lauren Azevedo, MS, DO^1^*, Rofida Nofal, MD, MS^2^, Allison Jay, MD^1^*, Adonis Lorenzana, MD^1^, Sioban Keel, MD^3^, Roshini S. Abraham, PhD^4^ , Marshall Horwitz, MD, PhD^3^, Michelle van Hee, BS^4^ , and Hadi Sawaf, MD^1^

^1^St John Providence Children’s Hospital, Detroit, MI, USA

^2^UCSF Beniof Children’s Hospital, Oakland, CA, USA

^3^University of Washington, Seattle, WA, USA

^4^Mayo Clinic, Rochester, MN, USA

* These authors contributed to the case report equally.

The revised Acknowledgments section follows:

**Acknowledgments**

Genetic testing for the patient was graciously performed at University of Washington under the direction of Dr Marshall Horwitz, Dr Sioban Keel, and Dr Mary-Claire King, and at the Mayo Clinic in Dr Roshini Abraham’s laboratory. The authors also wish to thank Dr Rofida Nofal for research into the background of GATA2.

